# Transforming Growth Factor‐β_1_ Modulates the Expression of Syndecan‐4 in Cultured Vascular Endothelial Cells in a Biphasic Manner

**DOI:** 10.1002/jcb.25861

**Published:** 2017-04-10

**Authors:** Takato Hara, Eiko Yoshida, Yasuyuki Fujiwara, Chika Yamamoto, Toshiyuki Kaji

**Affiliations:** ^1^ Faculty of Pharmaceutical SciencesDepartment of Environmental HealthTokyo University of ScienceNoda 278‐8510Japan; ^2^ Department of Environmental HealthSchool of PharmacyTokyo University of Pharmacy and Life SciencesHachioji 192‐0392Japan; ^3^ Faculty of Pharmaceutical SciencesDepartment of Environmental HealthToho UniversityFunabashi 274‐8510Japan

**Keywords:** ENDOTHELIAL CELL, PROTEOGLYCAN, SYNDECAN‐4, TGF‐β

## Abstract

Proteoglycans are macromolecules that consist of a core protein and one or more glycosaminoglycan side chains. Previously, we reported that transforming growth factor‐β_1_ (TGF‐β_1_) regulates the synthesis of a large heparan sulfate proteoglycan, perlecan, and a small leucine‐rich dermatan sulfate proteoglycan, biglycan, in vascular endothelial cells depending on cell density. Recently, we found that TGF‐β_1_ first upregulates and then downregulates the expression of syndecan‐4, a transmembrane heparan sulfate proteoglycan, via the TGF‐β receptor ALK5 in the cells. In order to identify the intracellular signal transduction pathway that mediates this modulation, bovine aortic endothelial cells were cultured and treated with TGF‐β_1_. Involvement of the downstream signaling pathways of ALK5—the Smad and MAPK pathways—in syndecan‐4 expression was examined using specific siRNAs and inhibitors. The data indicate that the Smad3–p38 MAPK pathway mediates the early upregulation of syndecan‐4 by TGF‐β_1_, whereas the late downregulation is mediated by the Smad2/3 pathway. Multiple modulations of proteoglycan synthesis may be involved in the regulation of vascular endothelial cell functions by TGF‐β_1_. J. Cell. Biochem. 118: 2009–2017,2017. © 2016 The Authors. *Journal of Cellular Biochemistry* Published by Wiley Periodicals, Inc.

Vascular endothelial cells cover the luminal surface of blood vessels in a monolayer and are thus a unique cell type because they have direct contact with blood. The cells regulate the blood coagulation‐fibrinolytic system by synthesizing and secreting several physiological substances, including von Willebrand factor [Jaffe et al., 1974], which facilitates blood coagulation; prostacyclin [Revtyak et al., 1987], which inhibits platelet aggregation; and plasminogen activator [Levin and Loskutoff, 1982], which converts plasminogen to plasmin, which then degrades the fibrin clot. Proteoglycans are macromolecules that consist of a core protein and glycosaminoglycan side chain(s) as a common feature and are a component of the extracellular matrix or are found on the cell surface. Although proteoglycans exhibit multiple functions such as extracellular matrix assembly, lipid metabolism, permeability and thrombosis [Camejo, 1981; Berenson et al., 1984] in vascular tissue, one of the most important functions appears to be the anticoagulant activity of glycosaminoglycan chains such as heparan sulfate and dermatan sulfate. The heparan sulfate and dermatan sulfate chains activate antithrombin III [Mertens et al., 1992] and heparin cofactor II [Tollefsen et al., 1983], respectively, and contribute to the anticoagulant property of vascular endothelium.

The major types of heparan sulfate proteoglycans synthesized by vascular endothelial cells are perlecan, which is the major extracellular matrix proteoglycan [Saku and Furthmayr, 1989]; members of the syndecan family of transmembrane proteoglycans such as syndecan‐1 and syndecan‐4 [Kojima et al., 1992]; and the cell surface‐associated proteoglycans such as glypican‐1 [Mertens et al., 1992]. The cells also synthesize small leucine‐rich dermatan sulfate proteoglycans such as biglycan and decorin [Järveläinen et al., 1991; Yamamoto et al., 2005]. It has been revealed that some cytokines and growth factors regulate proteoglycan synthesis in vascular endothelial cells. For example, fibroblast growth factor‐2 and vascular endothelial growth factor‐165 enhance the expression of biglycan and perlecan, respectively [Kinsella et al., 1997; Kaji et al., 2006]. Transforming growth factor‐β_1_ (TGF‐β_1_) induces the expression of biglycan and perlecan in a cell density‐dependent manner [Kaji et al., 2000]. In addition, connective tissue growth factor suppresses the synthesis of biglycan but newly induces the synthesis of decorin in the cells when cell density is low [Kaji et al., 2004].

Recently, we found that TGF‐β_1_ regulates the expression of syndecan‐4 in vascular endothelial cells in a biphasic manner [Hara et al., 2016]. The cytokine first upregulates and then downregulates endothelial syndecan‐4 expression. Two receptors for TGF‐β_1_ are expressed in vascular endothelial cells: activin receptor‐like kinase (ALK) 1 and ALK5 [Goumans et al., 2002; Shi and Massagué, 2003]. ALK1 activates its downstream transcriptional factor Smad1/5/8 [Goumans et al., 2003], whereas ALK5 can activate both the canonical Smad2/3 pathway and the non‐Smad pathway composed of mitogen‐activated protein kinases (MAPKs), including extracellular signal‐regulated kinase1/2 (ERK1/2), c‐jun N‐terminal kinase (JNK), and p38 MAPK [Derynck and Zhang, 2003; Moustakas and Heldin, 2005; Macias et al., 2015]. It is possible that the biphasic regulation of endothelial syndecan‐4 expression by TGF‐β_1_ is mediated by the Smad pathway, non‐Smad pathway, or both.

The present study used a culture system of bovine aortic endothelial cells to clarify the intracellular signal transduction pathways that mediates the early upregulation and the late downregulation of endothelial syndecan‐4 expression by TGF‐β_1_.

## MATERIALS AND METHODS

### MATERIALS

Vascular endothelial cells derived from bovine aorta were obtained from Cell Applications (San Diego, CA). Dulbecco's modified Eagle's medium and Ca^2+^‐ and Mg^2+^‐free phosphate‐buffered saline were obtained from Nissui Pharmaceutical (Tokyo, Japan). Tissue culture dishes and plates were obtained from Iwaki (Chiba, Japan). Fetal bovine serum was obtained from HyClone Laboratories (Waltham, MA). Recombinant human TGF‐β_1_ was purchased from Wako (Osaka, Japan). Heparinase II (derived from *Flavobacterium heparinum*), heparinase III (EC 4.2.2.8, derived from *Flavobacterium heparinum*), and diethylaminoethyl‐Sephacel (DEAE‐Sephacel) were purchased from Sigma‐Aldrich (St Louis, MO). PD98059, SP600125, and SB203580 were purchased from Cayman Chemical (Ann Arbor, MI). Goat polyclonal antibody against syndecan‐4 (N‐19) was obtained from Santa Cruz Biotechnology (Santa Cruz, CA). Anti‐Smad2/3 (#8685), anti‐phospho‐Smad2/3 (#8828), anti‐ERK1/2 (#9102), anti‐phospho‐ERK1/2 (#9101), anti‐JNK (#9252), anti‐phospho‐JNK (#9255), anti‐p38 MAPK (#9212), anti‐phospho‐p38 MAPK (#9211), horseradish peroxidase (HRP)‐conjugated anti‐rabbit (#7074), and HRP‐conjugated anti‐mouse (#7076) IgG antibodies were obtained from Cell Signaling Technology (Beverly, MA). HRP‐conjugated anti‐goat IgG antibody (ab6885) was obtained from Abcam (Bristol, UK). Mouse monoclonal antibody against GAPDH (015–25473) was purchased Wako Purechemical Industries (Osaka, Japan). Lipofectamine RNAiMAX and Opti‐MEM were obtained from Invitrogen (Carlsbad, CA). Other reagents, which were of the highest grade, were obtained from Nacalai Tesque (Kyoto, Japan).

### CELL CULTURE AND TREATMENT

Vascular endothelial cells were cultured in a humidified atmosphere of 5% CO_2_ at 37°C in Dulbecco's modified Eagle's medium supplemented with 10% fetal bovine serum until confluent. The cells were transfected with siRNAs for knockdown of Smad2, Smad3, or both and then stimulated with TGF‐β_1_ (1 or 5 ng/mL) for 6 or 24 h at 37°C. In another experiment, confluent cultures of the cells were treated with PD98059 (20 μM), SP600125 (10 μM), or SB203580 (10 μM) for 1 h and then stimulated with TGF‐β_1_ (5 ng/mL) for 6 or 24 h. The expression levels of syndecan‐4, ERK1/2, JNK, p38 MAPK, and Smad2/3 were determined by real‐time RT‐PCR or Western blot analysis as described below.

### siRNA TRANSFECTION

The transient transfection of siRNAs was performed using Lipofectamine RNAiMAX, according to the manufacturer's protocol. Briefly, an annealed siRNA duplex and Lipofectamine RNAiMAX were dissolved in Opti‐MEM in separate tubes and incubated for 5 min at room temperature. They were then mixed and incubated for 20 min at room temperature. Vascular endothelial cells were grown to about 80% confluence in Dulbecco's modified Eagle medium supplemented with 10% fetal bovine serum and incubated for 24 h at 37°C in fresh Dulbecco's modified Eagle medium supplemented with both 10% fetal bovine serum and the siRNA/Lipofectamine RNAiMAX mixture. The final concentrations of siRNA and Lipofectamine RNAiMAX were 40 nM and 0.2%, respectively. The siRNAs were purchased from FASMAC (Kanagawa, Japan) and the sequences of the sense and antisense strands of the siRNAs were as follows: bovine Smad2 siRNA (siSmad2), 5′‐UUCAAAACCCUGAUUAACGdTdT‐3′ (sense) and 5′‐CGUUAAUCAGGGUUUUGAAdTdT‐3′ (antisense), and bovine Smad3 siRNA (siSmad3), 5′‐UGUUUUCGGGGAUGGAAUGdTdT‐3′ (sense) and 5′‐CAUUCCAUCCCCGAAAACAdTdT‐3′ (antisense). Negative control siRNA (siControl) (Qiagen, Valencia, CA) was used as a nonspecific sequence.

### REAL‐TIME RT‐PCR

A monolayer of vascular endothelial cells was washed twice with Ca^2+^‐ and Mg^2+^‐free phosphate‐buffered saline and lysed with QIAzol lysis reagent (Qiagen). A quarter volume of chloroform was mixed with the lysate, and the mixture was centrifuged. The supernatant was collected, 70% ethanol was added to a concentration of 52.5%, the suspension was centrifuged at 20,000 × *g*, and the supernatant was discarded. The precipitate was suspended again in 70% ethanol and centrifuged at 20,000 × *g*, and the obtained precipitate containing the total RNA was dried. Complementary DNA was synthesized from the mRNA using a high‐capacity cDNA reverse transcription kit (Applied Biosystems, Foster, CA). Real‐time PCR was performed using GeneAce SYBR qPCR Mix α (Nippon Gene, Tokyo, Japan) with 1 ng/μL cDNA and 0.1 μM primers on a StepOnePlus Real‐Time PCR System (Applied Biosystems). The levels of syndecan‐4 and glyceraldehyde 3‐phosphate dehydrogenase (GAPDH) mRNAs were quantified by the relative standard curve method. The fold change of the intensity value of syndecan‐4 was normalized by that of GAPDH. The sequences of the bovine gene‐specific forward and reverse primers were as follows: syndecan‐4, 5′‐TTGCCGTCTTCCTCGTGC‐3′ (forward) and 5′‐AGGCGTAGAACTCATTGGTGG‐3′ (reverse), Smad2, 5′‐CAGAATACCGAAGGCAGACG‐3′ (forward) and 5′‐ TGAGCAACGCACTGAAGG −3′ (reverse), Smad3, 5′‐ACTACAGCCATTCCATCC‐3′ (forward) and 5′‐ATCTGGTGGTCACTGGTCTC‐3′ (reverse), TSP‐1, 5′–3′ (forward) and 5′–3′ (reverse), PAI‐1, 5′‐CCGTGGAACAAGGATGAG‐3′ (forward) and 5′‐ CGGAACAGCCTGAAGAAG‐3′ (reverse), and GAPDH, 5′‐AACACCCTCAAGATTGTCAGCAA‐3′ (forward) and 5′‐ACAGTCTTCTGGGTGGCAGTGA −3′(reverse).

### PROTEOGLYCAN CORE PROTEIN EXTRACTION AND WESTERN BLOT ANALYSIS

Proteoglycans were extracted from the cell layer and the conditioned medium of vascular endothelial cells under dissociative conditions. Specifically, the conditioned medium was harvested, and solid urea was added to a final concentration of 8 M. The cell layer was washed twice with Ca^2+^‐ and Mg^2+^‐free phosphate‐buffered saline and lysed with 8 M urea cell extract solution (pH 7.5) containing 120 mM 6‐aminohexanoic acid, 12 mM benzamidine, 10 mM N‐ethylmaleimide, 2 mM EDTA, 0.1 M phenylmethanesulfonyl fluoride, 0.1 M NaCl, 50 mM Tris base, and 2% Triton X‐100. The extracts were applied to DEAE‐Sephacel (0.3 mL of resin) columns and washed four times with 8 M urea buffer (pH 7.5) containing 0.25 M NaCl, 2 mM EDTA, 0.5% Triton X‐100, and 50 mM Tris base. Proteoglycans were eluted with 0.9 mL 3 M urea buffer (pH 7.5) containing 2 mM EDTA, 0.5% Triton X‐100, and 50 mM Tris base and precipitated with 3.5 volumes of 1.3% potassium acetate in 95% ethanol for 2 h at −20°C; this precipitation step was repeated three times. Precipitated proteoglycans were digested with heparinase II and III (13.3 U/mL each) in 100 mM Tris‐HCl buffer (pH 7.0) containing 10 mM calcium acetate and 18 mM sodium acetate for 3 h at 37°C to determine core proteins of syndecan‐4. Separately, total proteins from vascular endothelial cells were prepared by lysis in sodium dodecyl sulfate (SDS) sample buffer (50 mM Tris–HCl buffer solution containing 2% SDS and 10% glycerol, pH 6.8) followed by incubation at 95°C for 10 min. The protein concentration was determined using a BCA protein assay kit (Thermo Scientific, Waltham, MA) before adding 2‐mercaptoethanol and bromophenol blue to the samples. The proteoglycans and cellular proteins were separated by SDS‐polyacrylamide gel electrophoresis on a 10% polyacrylamide gel and then transferred to a polyvinyl difluoride membrane (Immobilon‐P, Millipore, Billerica, MA) at 2 mA/cm^2^ for 1 h following the method of Kyhse‐Andersen [Kyhse‐Andersen, 1984]. Membranes were blocked with 2% BSA solution or 5% skim milk in 20 mM Tris‐HCl buffer solution (pH 7.5) containing 150 mM NaCl and 0.1% Tween 20 and then incubated overnight with a primary antibody against syndecan‐4, Smad2/3, phosphorylated Smad2/3, ERK1/2, phosphorylated ERK1/2, JNK, phosphorylated JNK, p38 MAPK, or phosphorylated p38 MAPK at 4°C. The membranes were washed and then incubated with HRP‐conjugated secondary antibodies for 1 h at room temperature. Immunoreactive bands were visualized using enhanced chemiluminescence western blotting detection reagents (Chemi‐Lumi One L, Nacalai, Kyoto, Japan) and scanned with a LAS 3000 Imager (Fujifilm, Tokyo, Japan). Representative blots from three independent experiments are shown.

### STATISTICAL ANALYSIS

Data were analyzed for statistical significance by analysis of variance and Student's *t*‐test or Tukey's method, as appropriate. *P* values of less than 0.05 were considered statistically significantly different.

## RESULTS

### TGF‐β_1_ ACTIVATES p38 MAPK AND Smad2/3 IN VASCULAR ENDOTHELIAL CELLS

**Figure 1:**
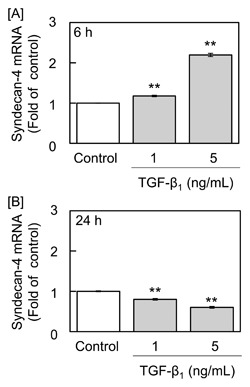
Effects of TGF‐β_1_ on the expression of syndecan‐4 mRNA in vascular endothelial cells. Bovine aortic endothelial cells were treated with 1 and 5 ng/mL TGF‐β_1_ at 37°C for 6 or 24 h ([A] and [B], respectively). Values are the mean ± S.E. of four samples. Significantly different from the corresponding control, **P < 0.01.

Figure 1 shows the expression of syndecan‐4 mRNA in vascular endothelial cells treated with TGF‐β_1_. The expression of syndecan‐4 mRNA was elevated at 6 h and reduced at 24 h by the cytokine at 1 and 5 ng/mL. This result is consistent with our recent study [Hara et al., 2016], showing that TGF‐β_1_ modulates endothelial syndecan‐4 expression in a biphasic manner.

**Figure 2:**
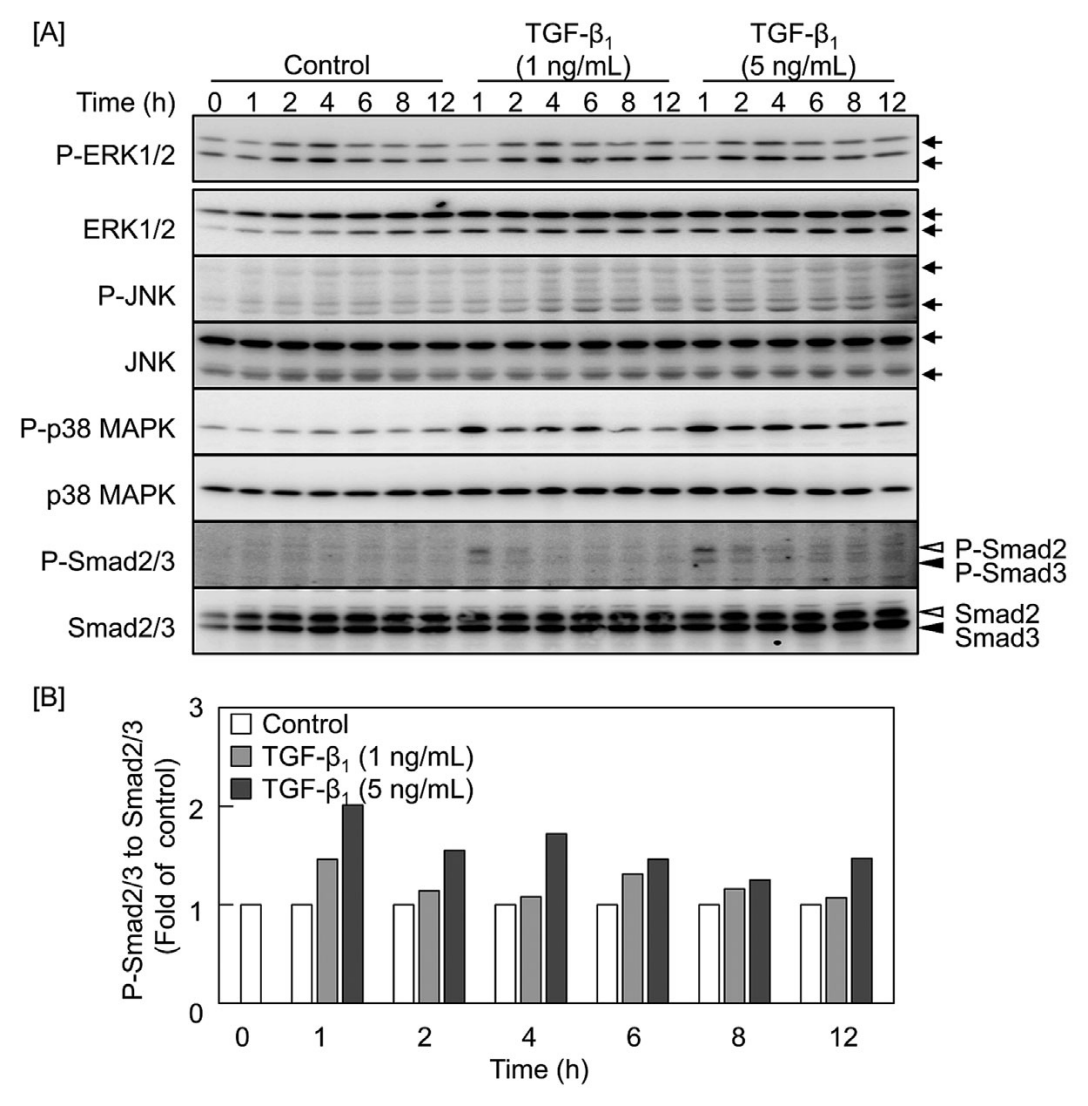
Effects of TGF‐β_1_ on the activation of ERK1/2, JNK, p38 MAPK, and Smad2/3 in vascular endothelial cells. Bovine aortic endothelial cells were treated with 1 and 5 ng/mL TGF‐β_1_ at 37°C for 1, 2, 4, 6, 8, or 12 h. [A] Expression of P‐ERK1/2, ERK1/2, P‐JNK, JNK, P‐p38 MAPK, p38 MAPK, P‐Smad2/3, and Smad2/3 proteins. Open and filled arrowheads indicate the position of Smad2 and Smad3, respectively. Arrows indicate the positions of P‐ERK1/2, ERK1/2, P‐JNK, and JNK. [B] The ratio of the intensity of P‐Smad2/3 to that of Smad2/3 in [A].

Figure 2 shows the phosphorylation of MAPKs (ERK1/2, JNK, and p38 MAPK) and Smad2/3, which may be involved in the modulation of syndecan‐4 expression by TGF‐β_1_ as the downstream signaling pathways of the cytokine. For the MAPKs, the phosphorylation of only p38 MAPK was elevated by 1 and 5 ng/mL TGF‐β_1_ after 1 h and longer. On the other hand, the phosphorylation of Smad2/3 was increased by 1 ng/mL TGF‐β_1_ after 1h and the increase disappeared after 2 h and longer. TGF‐β_1_ at 5 ng/mL the phosphorylation of Smad2/3 after 1 h and longer.

### THE p38 MAPK PATHWAY MEDIATES THE EARLY UPREGULATION OF ENDOTHELIAL SYNDECAN‐4 EXPRESSION BY TGF‐β_1_

**Figure 3:**
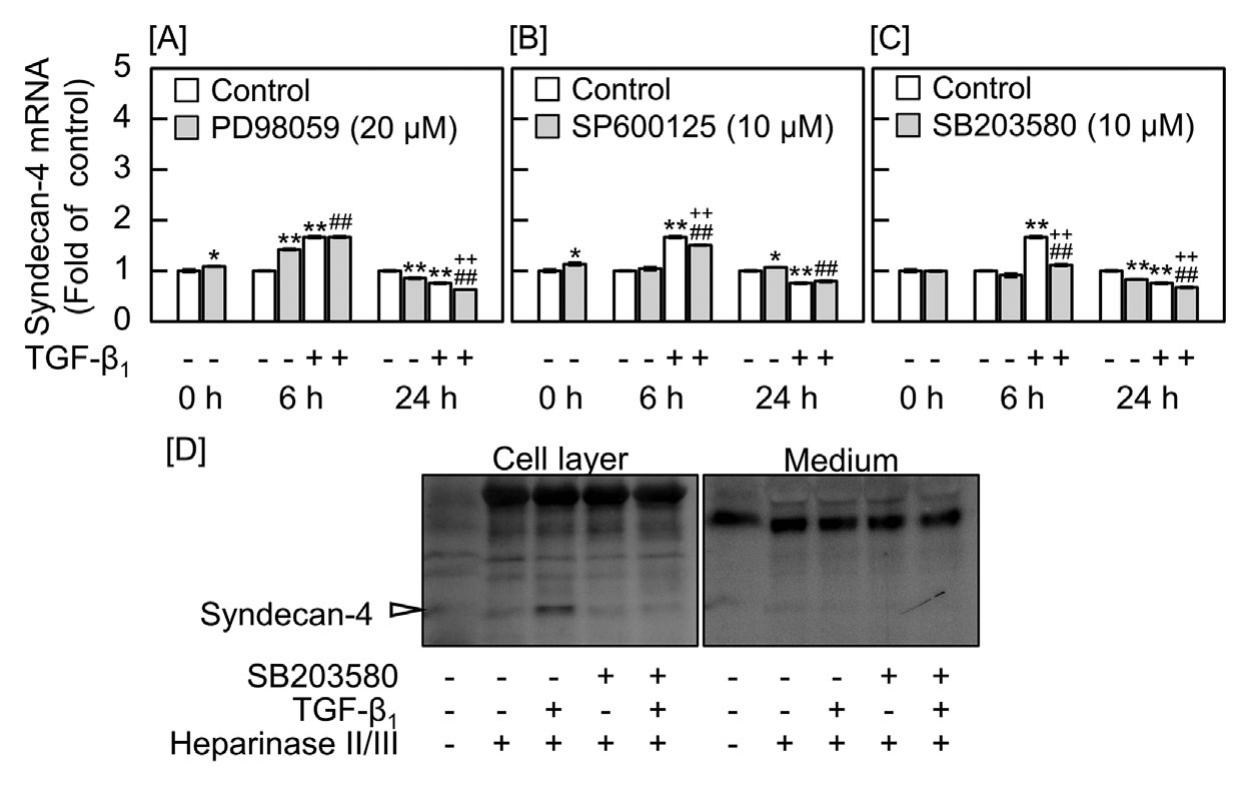
Effects of the MAPK pathway inhibitors PD98059, LY364947, and SB203580 on the expression of syndecan‐4 in vascular endothelial cells. Bovine aortic endothelial cells were pretreated with [A] the MEK1 inhibitor PD98059 at 20 μM, [B] JNK inhibitor SP600125 at 10 μM, or [C] p38 MAPK inhibitor SB203580 at 10 μM at 37°C for 1 h and then treated with 5 ng/mL TGF‐β_1_ for 6 or 24 h. Values are the mean ± S.E. of four samples. *P < 0.05, **P < 0.01 versus control; *^−^P* < 0.01 versus MAPK inhibitor without TGF‐β_1_; ^++^ *P* < 0.01 versus TGF‐β_1_. [D] The expression of syndecan‐4 core protein. Arrowhead indicates the position of syndecan‐4. Bovine aortic endothelial cells were pretreated with 10 μM SB203580 at 37°C for 1 h and then treated with 5 ng/mL TGF‐β_1_ for 6 h.

To examine the involvement of MAPKs in the modulation of syndecan‐4 mRNA expression by TGF‐β_1_, vascular endothelial cells were pretreated with the MEK1 inhibitor PD98059, JNK inhibitor SP600125, or p38 MAPK inhibitor SB203580 and then stimulated with TGF‐β_1_ (Fig. 3). Syndecan‐4 mRNA expression was upregulated after 6 h and downregulated after 24 h by TGF‐β_1_. PD98059 (Fig. 3A) and SP600125 (Fig. 3B) did not influence the early upregulation or the late downregulation. In contrast, SB203580 suppressed the early upregulation of syndecan‐4 mRNA expression by TGF‐β_1_, although the late downregulation was unaffected by the inhibitor (Fig. 3C). In addition, TGF‐β_1_ increased syndecan‐4 core protein expression in the cell layer, and this increase was completely diminished by SB203580 (Fig. 3D); the core protein was not detected in the conditioned medium. The intensity of nonspecific bands was almost same after treatment with heparinase II/III. These results suggest that TGF‐β_1_ activates p38 MAPK, which mediates the early upregulation of syndecan‐4 expression by the cytokine in vascular endothelial cells.

### Smad2/3 PATHWAYS MEDIATE THE LATE DOWNREGULATION OF SYNDECAN‐4 EXPRESSION BY TGF‐β_1_

**Figure 4:**
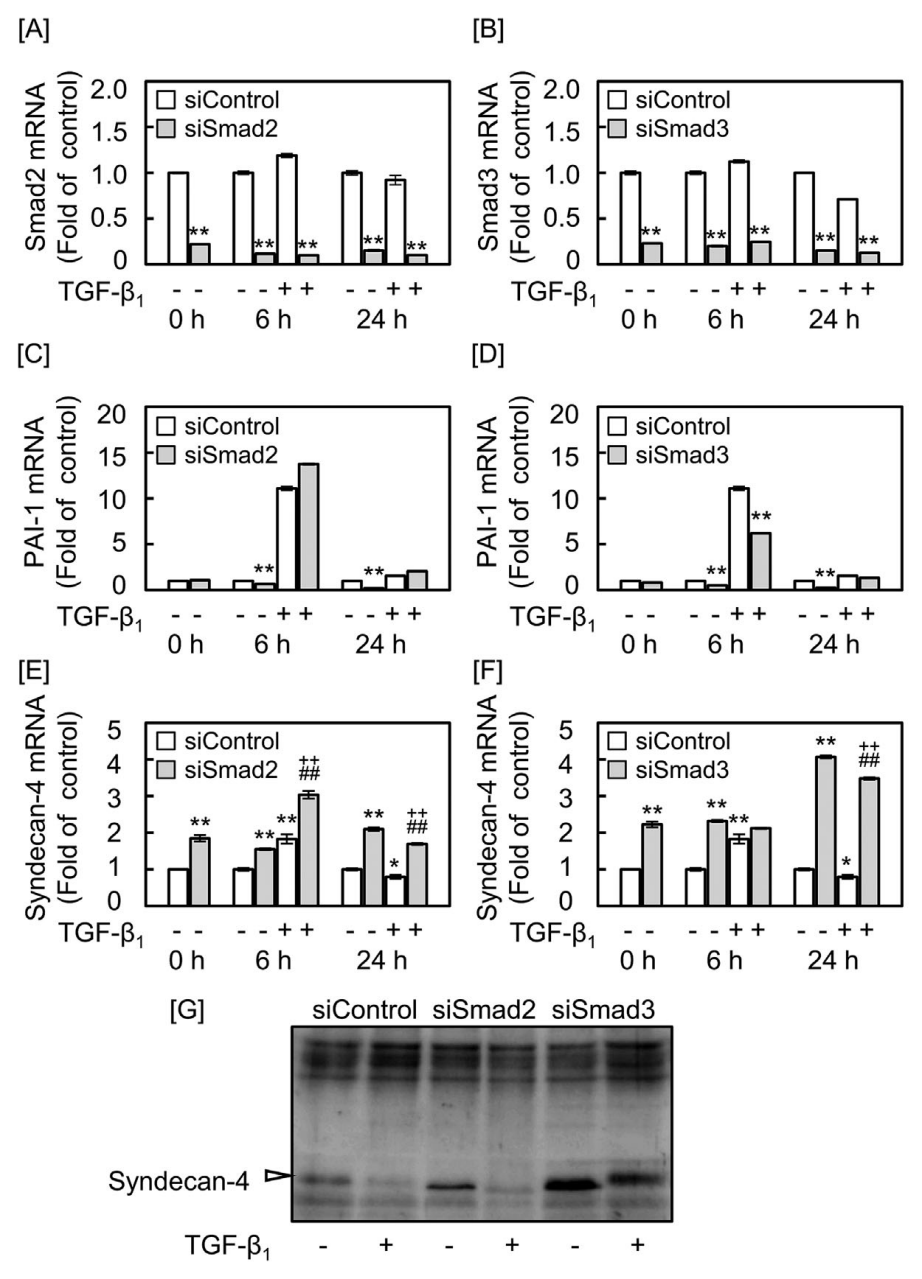
Effects of siRNA‐mediated knockdown of Smad2 or Smad3 on the expression of syndecan‐4 in vascular endothelial cells. Bovine aortic endothelial cells were transfected with siSmad2 or siSmad3 at 37°C for 24 h and then treated with 5 ng/mL TGF‐β_1_ for 6 or 24 h. The expression of mRNAs for [A] Smad2, [B] Smad3, [C and D] PAI‐1, and [E and F] syndecan‐4. Values are the means ± S.E. of four samples. *P < 0.05, **P < 0.01 versus siControl; ^*−*^ *P* < 0.01 versus siSmad2 or siSmad3 without TGF‐β_1_; ^++^ *P* < 0.01 versus TGF‐β_1_. [G] The expression of syndecan‐4 core protein. Arrowhead indicates the position of syndecan‐4. Bovine aortic endothelial cells were transfected with siControl, siSmad2, or siSmad3 at 37°C for 24 h and then treated with 5 ng/mL TGF‐β_1_ for 24 h.

To examine the involvement of Smad2 and Smad3 in the modulation of syndecan‐4 expression by TGF‐β_1_, vascular endothelial cells were transfected with siSmad2 or siSmad3 and then treated with 5 ng/mL TGF‐β_1_ (Fig. 4). The expression of Smad2 (Fig. 4A) and Smad3 (Fig. 4B) was markedly reduced by treatment with siSmad2 and siSmad3, respectively. Additionally, we measured the expression of plasminogen activator inhibitor‐1 (PAI‐1) mRNA to confirm the functional knockdown of Smad2 and Smad3 (Figs. 4C and 4D). After a 24‐h transfection of either siSmad2 or siSmad3 in the absence of TGF‐β_1_, the PAI‐1 mRNA expression was markedly reduced. This suggests that the basal level of PAI‐1 mRNA was suppressed by either Smad2 or Smad3 knockdown. After a 6‐h incubation with TGF‐β_1_, the PAI‐1 mRNA expression was markedly induced; siSmad3 but not siSmad2 significantly suppressed the induction, suggesting that TGF‐β_1_‐induced PAI‐1 mRNA expression was suppressed only by siSmad3. These results were consistent with previous reports [Yingling et al., 1997; Xu et al., 2000] and showed a specific knockdown of Smad2 and Smad3 by siSmad2 and siSmad3, respectively. siRNA‐mediated knockdown alone of either Smad2 (Fig. 4E) or Smad3 (Fig. 4F) significantly increased the expression of endothelial syndecan‐4 mRNA without treatment with TGF‐β_1_, suggesting that both Smad2 and Smad3 are involved in the reduction of the basal expression of endothelial syndecan‐4. The early upregulation and late downregulation of syndecan‐4 mRNA expression by TGF‐β_1_ were both not affected by siRNA‐mediated knockdown of Smad2 (Fig. 4E). On the other hand, siRNA‐mediated knockdown of Smad3 diminished the upregulation of syndecan‐4 mRNA expression by TGF‐β_1_ (Fig. 4F), suggesting that Smad3 is a member of the signaling pathway that mediates the TGF‐β_1_‐induced upregulation of syndecan‐4 expression. The late downregulation of syndecan‐4 core protein expression by TGF‐β_1_ was observed in cells treated with or without siRNA for Smad2, but this downregulation was reduced by siRNA for Smad3 (Fig. 4G). This result indicates that Smad3 is involved in the late downregulation of syndecan‐4 core protein expression by TGF‐β_1_. These results indicate that the early upregulation of syndecan‐4 mRNA expression by TGF‐β_1_ is mediated by Smad3, whereas Smad2 or Smad3 alone is not involved in the late downregulation of syndecan‐4 mRNA expression by TGF‐β_1_; the late downregulation of syndecan‐4 core protein expression by TGF‐β_1_ is mediated by Smad3.

To clarify the role of Smad2/3 in the late downregulation, the effects of the knockdown of both Smad2 and Smad3 on the expression of syndecan‐4 mRNA and the core protein were investigated. The results indicated that the siRNA‐mediated knockdown of both Smad2 and Smad3 diminished the late downregulation of syndecan‐4 mRNA expression by TGF‐β_1_, although the knockdown of just one could not diminish the downregulation (Fig. S1, upper panel). In addition, the results again indicated that siSmad3 with or without siSmad2 diminished the decrease in syndecan‐4 core protein expression by TGF‐β_1_ after a 24‐h treatment (Fig. S1, lower panel). These results suggest that the late downregulation of syndecan‐4 expression by TGF‐β_1_ is mediated by Smad2/3 at the mRNA level and by Smad3 at the protein level.

### ACTIVATION OF p38 MAPK IS MEDIATED BY Smad3

**Figure 5:**
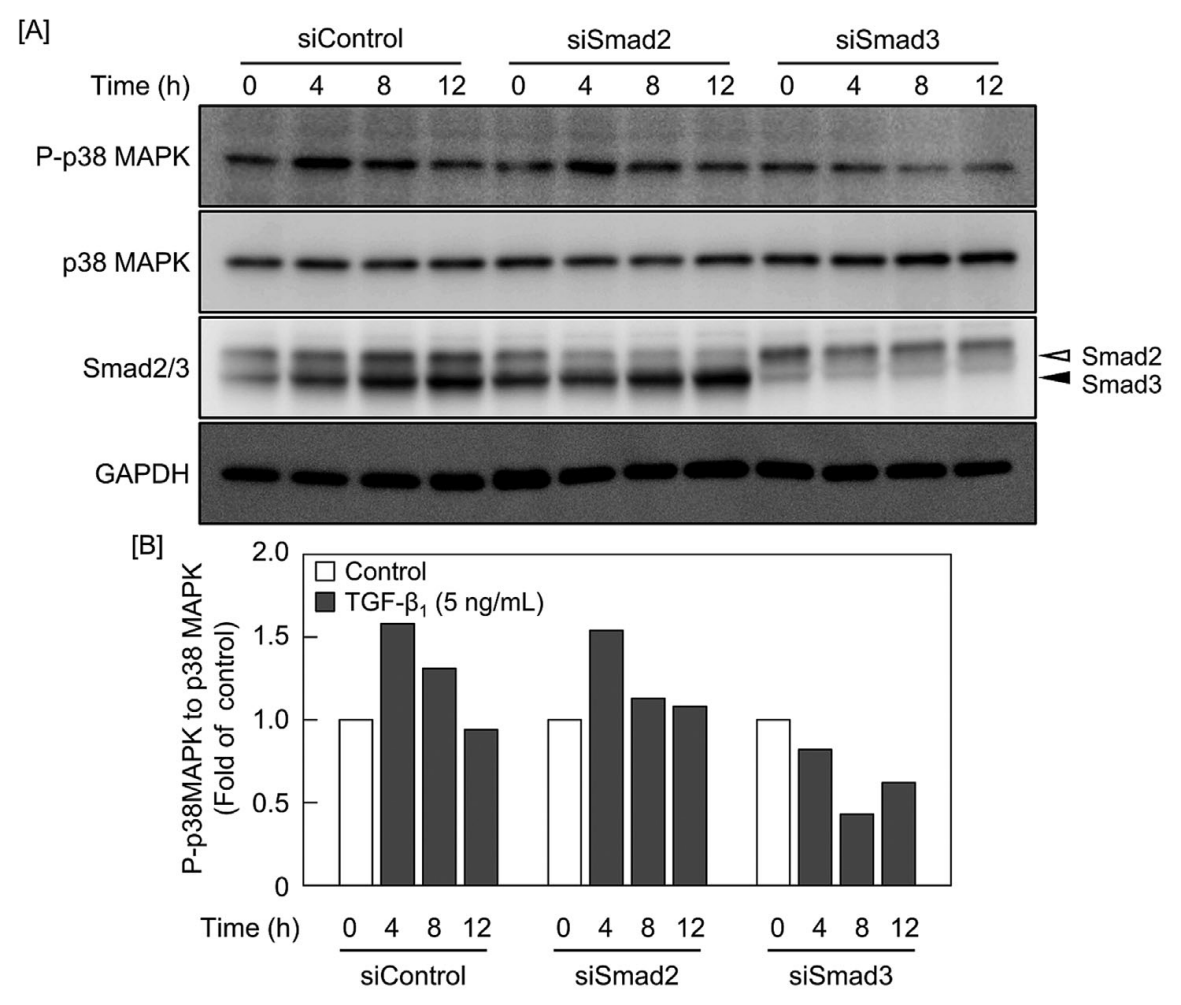
Effects of siRNA‐mediated knockdown of Smad2 or Smad3 on the activation of p38 MAPK in vascular endothelial cells. Bovine aortic endothelial cells were transfected with siSmad2 or siSmad3 at 37°C for 24 h and then treated with 5 ng/mL TGF‐β_1_ for 4, 8, or 12 h. [A] Expression of P‐p38 MAPK, p38 MAPK, Smad2/3, and GAPDH proteins. Open and filled arrowheads indicate the position of Smad2 and Smad3, respectively. [B] The ratio of the intensity of P‐p38 MAPK to that of p38 MAPK in [A].

In order to examine whether the phosphorylation of p38 MAPK by TGF‐β_1_ is induced by Smad2 or Smad3, vascular endothelial cells transfected with siSmad2 or siSmad3 were stimulated with TGF‐β_1_, and the phosphorylation of p38 MAPK was investigated. As shown in Figure 5, TGF‐β_1_ increased the phosphorylation of p38 MAPK after 4 h and 8 h; the increase was markedly suppressed by siSmad3 but not siSmad2, suggesting that the activation of p38 MAPK is mediated by Smad3.

## DISCUSSION

Recently, we reported that biglycan intensifies the ALK5‐Smad2/3 signaling induced by TGF‐β_1_ and downregulates syndecan‐4 in cultured vascular endothelial cells [Hara et al., 2016]. In this study, we observed that TGF‐β_1_ first upregulates and then downregulates endothelial syndecan‐4 expression. In the present study, we investigated the signaling pathways that mediate the biphasic regulation of endothelial syndecan‐4 expression, and the following results were obtained: (1) The biphasic regulation by TGF‐β_1_ was confirmed. (2) TGF‐β_1_ activated MAPKs, including ERK1/2, JNK, and p38 MAPK, as well as Smad2/3. (3) The early upregulation of syndecan‐4 by TGF‐β_1_ was diminished by either an inhibitor of p38 MAPK or siRNA for Smad3 at both the mRNA and syndecan‐4 core protein expression levels. (4) The late downregulation of syndecan‐4 mRNA expression was reduced when cells were treated with siRNAs for both Smad2 and Smad3, whereas the late downregulation of syndecan‐4 core protein expression was reduced only by siRNA for Smad3. (5) The activation of p38 MAPK was reduced by siRNA for Smad3 but not by that for Smad2. Together, these results suggest that the early upregulation of endothelial syndecan‐4 expression by TGF‐β_1_ is mediated by the ALK5–Smad3–p38 MAPK pathway, whereas the late downregulation is mediated by the ALK5–Smad2/3 pathway. Specifically, the downregulation of syndecan‐4 expression by TGF‐β_1_ is mediated by the interaction of Smad3 with Smad2 at the mRNA expression level, whereas only Smad3 is crucial at the core protein expression level. The present data showed for the first time that TGF‐β_1_ and p38 MAPK serve as regulatory molecules for endothelial syndecan‐4 expression.

The TGF‐β_1_ that vascular endothelial cells are exposed to is mainly released from aggregated platelets. When vascular endothelial cells are injured, platelets aggregate at the damaged site, and TGF‐β_1_ is released from α‐granules in the platelets [Assoian and Sporn, 1986]. At that time, TGF‐β_1_ activates p38 MAPK and upregulates plasminogen activator inhibitor type 1 expression to maintain the fibrin clot that prevents bleeding [Woodward et al., 2006]. TGF‐β_1_‐activated p38 MAPK increases permeability [Goldberg et al., 2002] and induces the apoptosis of vascular endothelial cells [Hyman et al., 2002]. In addition to these inflammatory responses, the TGF‐β_1_–p38 MAPK pathway also increases the synthesis and activity of focal adhesion kinase, which is crucial for cell survival, motility, and proliferation [Walsh et al., 2008]. Syndecan‐4 molecules have three heparan sulfate chains on the extracellular domain of their core proteins that are used for focal adhesion; the heparan sulfate chains play a critical role in stress fiber formation [Gopal et al., 2010]. It has also been reported that syndecan‐4 is an anti‐inflammatory molecule. Specifically, a large number of neutrophils, increased expression of neutrophil chemotactic factors, and higher mortality were reported in syndecan‐4 knockout mice injected with lipopolysaccharide [Ishiguro et al., 2001; Tanino et al., 2012]. These results and our data, which showed that the induction of endothelial syndecan‐4 expression occurs in the early stage of the exposure to TGF‐β_1_, suggest that the upregulation of endothelial syndecan‐4 expression by TGF‐β_1_ may be a defense mechanism against an acute injury to vascular endothelial cells.

It has been reported that the atherosclerotic vascular wall has fewer heparan sulfate proteoglycans and more dermatan sulfate proteoglycans compared to the normal vascular wall [Stevens et al., 1976]. In addition, the lack of syndecan‐4 disrupts the alignment of vascular endothelial cells along the direction of blood flow, resulting in the formation of a wide range atherosclerotic lesions, including near vascular branching points where jet laminar flow is impeded to create disturbed flow [Baeyens et al., 2014]. The disturbed flow increases thrombogenicity and triggers chronic inflammation [Brooks et al., 2002]. Additionally, vascular endothelial cells exposed to disturbed flow exhibit a lower expression of focal adhesion molecules such as VE‐cadherin and β‐catenin than cells exposed to pulsatile flow [Miao et al., 2005]. The functional damage of vascular endothelial cells caused by the disturbed flow induces chronic TGF‐β_1_ accumulation in the vascular wall, and the accumulated TGF‐β_1_ suppresses the expression of syndecan‐4 by activation of Smad2/3, which contributes to the progression of atherosclerosis [Tull et al., 2006; Nesbitt et al., 2009; Popovic et al., 2009]. Together with the results from our previous study, it is suggested that biglycan, a proteoglycan whose synthesis is induced by TGF‐β_1_ [Kaji et al., 2000], intensifies the TGF‐β_1_–ALK5 signaling via the core protein and that activated Smad2/3 in the signaling pathway suppresses the expression of syndecan‐4 at the late stage of TGF‐β_1_ exposure in vascular endothelial cells. In other words, TGF‐β_1_ induces endothelial biglycan synthesis; as a result, syndecan‐4 expression is suppressed by enhanced TGF‐β_1_ signaling. This regulation of endothelial proteoglycan synthesis by TGF‐β_1_ may be a component of the mechanisms underlying the histopathological changes in the types of proteoglycans—fewer heparan sulfate proteoglycans and more dermatan sulfate proteoglycans [Stevens et al., 1976]—seen in atherosclerotic vascular walls.

The data from the present study revealed the signaling pathways that mediate the regulation of syndecan‐4 expression by TGF‐β_1_ in vascular endothelial cells. They indicated that TGF‐β_1_ upregulates syndecan‐4 expression via the ALK5–Smad3–p38 MAPK pathway at the early stage, whereas downregulation of the expression occurs via the ALK5–Smad2/3 pathway. The mechanism responsible for switching the signaling pathway from the Smad3–p38 MAPK pathway to the Smad2/3 pathway for syndecan‐4 expression appears to be important. It was reported that the signal transduction of Ask1, which is a MAP kinase kinase kinase that regulates the stress responses, is regulated in a biphasic manner by ubiquitination. Ubiquitinated Ask1 is transiently activated and transduces a proliferation signal [Maruyama et al., 2014]; however, when Ask1 is not ubiquitinated, the molecule is activated continuously and induces an apoptotic signal [Ichijo et al., 1997]. Because Smad3 is also ubiquitinated [Fukuchi et al., 2001; Moren et al., 2003], it may be possible that one of the mechanisms that regulates the signaling pathway for endothelial syndecan‐4 is the ubiquitination of Smad3. Further studies are required to clarify the detailed mechanisms by which the signaling pathway for endothelial syndecan‐4 expression is switched from the Smad3–p38 MAPK pathway for upregulation at the early stage to the Smad2/3 pathway for downregulation at the late stage.

## Supporting information

Additional supporting information may be found in the online version of this article at the publisher's web‐site.

Supporting Figure S1.
