# Supplementary figures and images for: Transforming Growth Factor‐β1 Modulates the Expression of Syndecan‐4 in Cultured Vascular Endothelial Cells in a Biphasic Manner
Source: J Cell Biochem. 2017 Apr 10;118(8):2009–17. doi: 10.1002/jcb.25861 (PMC5485002; doi:10.1002/jcb.25861)

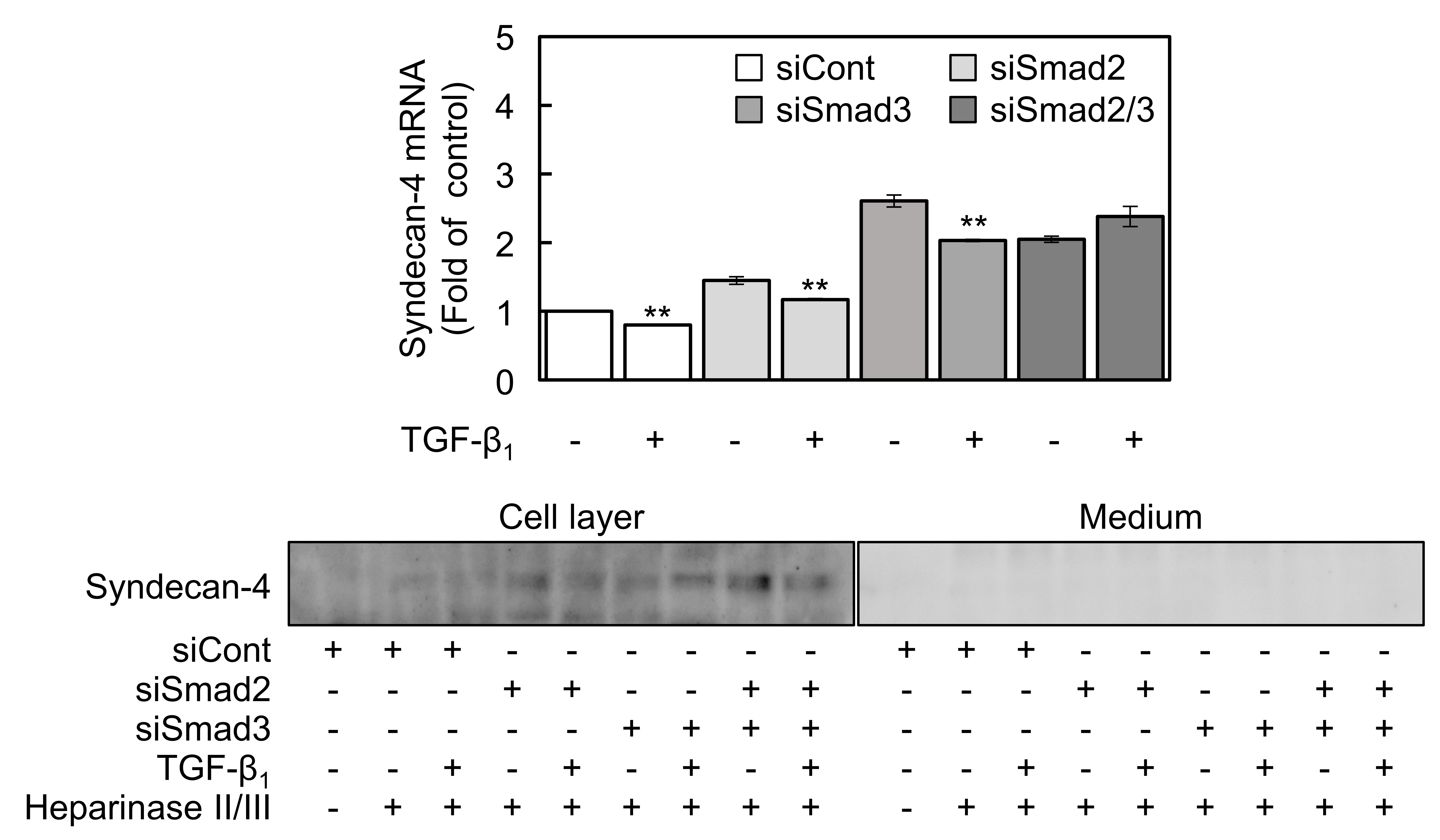

Supplement: Supplementary file 1 — Supporting Figure S1. [file JCB-118-2009-s001.tif]
